# RhoA Proteolysis Regulates the Actin Cytoskeleton in Response to Oxidative Stress

**DOI:** 10.1371/journal.pone.0168641

**Published:** 2016-12-19

**Authors:** Marie-Pier Girouard, Madeline Pool, Ricardo Alchini, Isabel Rambaldi, Alyson E. Fournier

**Affiliations:** Department of Neurology and Neurosurgery, Montreal Neurological Institute, Rue University, Montréal, Québec, Canada; Texas A&M University Health Sciences Center, UNITED STATES

## Abstract

The small GTPase RhoA regulates the actin cytoskeleton to affect multiple cellular processes including endocytosis, migration and adhesion. RhoA activity is tightly regulated through several mechanisms including GDP/GTP cycling, phosphorylation, glycosylation and prenylation. Previous reports have also reported that cleavage of the carboxy-terminus inactivates RhoA. Here, we describe a novel mechanism of RhoA proteolysis that generates a stable amino-terminal RhoA fragment (RhoA-NTF). RhoA-NTF is detectable in healthy cells and tissues and is upregulated following cell stress. Overexpression of either RhoA-NTF or the carboxy-terminal RhoA cleavage fragment (RhoA-CTF) induces the formation of disorganized actin stress fibres. RhoA-CTF also promotes the formation of disorganized actin stress fibres and nuclear actin rods. Both fragments disrupt the organization of actin stress fibres formed by endogenous RhoA. Together, our findings describe a novel RhoA regulatory mechanism.

## Introduction

RhoA is a ubiquitously expressed member of the Ras-related family of GTPases with important roles in the regulation of the cytoskeleton through the assembly of actin stress fibres [1, 2] and the stabilization of microtubules [3, 4]. These effects on the cytoskeleton underlie important roles for RhoA in many cellular functions such as cytokinesis [5], adhesion [1, 2], and migration [6, 7]. The activation of RhoA is tightly regulated by several mechanisms. Cycling of RhoA between its inactive GDP-bound and active GTP-bound state is coordinated by GTPase activating proteins (GAPs) and guanine nucleotide exchange factors (GEFs) [8, 9]. In addition, guanine nucleotide dissociation inhibitors (GDIs) sequester RhoA in the cytosol and maintain it in its inactive GDP-bound state by interacting with the lipophilic moiety added post-translationally to the carboxy-terminal end of RhoA in a process known as prenylation [10–12]. Upon activation, RhoA is released from the GDIs and is able to translocate to the plasma membrane where it interacts with its numerous downstream effectors [12]. Additionally, RhoA is negatively regulated by phosphorylation of residue S188 by both cAMP- and cGMP-dependent kinases [13–16] as well as by tyrosine glycosylation of residue Y34 [17]. Thus, the combined effects of regulatory proteins and post-translational modifications control the activation of RhoA and, consequently, its functions.

The temporal and spatial functions of RhoA are also dependent on its stability. Thus, dynamic regulation through proteasomal degradation is critical for cell migration and the establishment of polarity. In protrusions, the Smurf1 E3 ubiquitin ligase controls the local level of RhoA by targeting it for proteasomal degradation [18]. The Skp1-Cullin1-F-box protein (SCF)-like E3 ubiquitin ligase complex acts similarly to regulate the formation of actin stress fibres and cell morphology [19, 20]. Additionally, during cytokinesis, active GTP-bound RhoA is degraded in autophagosomes at the cleavage furrow to limit its functions [21]. Moreover, proteolysis is another mechanism resulting in RhoA downregulation. The mammalian cysteine protease μ-calpain cleaves the carboxy-terminal end of RhoA, generating a dominant-negative protein that inhibits integrin-dependent cell spreading and actin stress fibre formation [22]. Similarly, upon infection by *Yersinia enterocolitica*, YopT (*Yersinia* outer protein), a cysteine protease expressed in host cells, inactivates RhoA by releasing it from the membrane and preventing its interaction with downstream effectors [23, 24]. Thus, several mechanisms contribute to the localized activity of RhoA.

Here, we describe a novel cleavage of RhoA that generates a stable amino-terminal cleavage fragment (RhoA-NTF). Cleavage occurs between the Switch I and Switch II regions and is regulated by the activity of serine proteases, calpain and caspases. The cleavage is upregulated in response to oxidative stress. High levels of RhoA-NTF or carboxy-terminal fragments (RhoA-CTF), such as those potentially found in pathological condition, induce the formation of actin stress fibres in the cytoplasm and RhoA-CTF also induces formation of small nuclear actin rods. Thus, we describe a novel mechanism of RhoA proteolysis that regulates the actin cytoskeleton in response to oxidative stress.

## Material and Methods

### Plasmids and cloning

Full-length untagged (clone ID: RHO0A00000) and 2Xmyc-tagged (clone ID: RHO0A0MN00) wild-type (WT) RhoA construct were obtained from the cDNA Resource Center (cDNA.org). To generate amino-terminal Flag-tagged constructs, the appropriate DNA was subcloned by PCR into a pcDNA3-Flag vector provided by Peter McPherson (Montreal Neurological Institute, Montréal, QC, Canada). A similar cloning was done to generate pGEX 4T-1 GST-tagged WT-RhoA. Other mutations (C190A, A56G/Q63L, A56V/Q63L, L57A/Q63L, W58A/Q63L, D59A/Q63L, Q63L/C83A, LWD-AAA/Q63L, Q63L/C190A, T19N/C190A) were generated by site-directed mutagenesis using the QuikChange II XL Site-Directed Mutagenesis Kit (Agilent Technologies, Santa Clara, CA). The coding sequences corresponding to the amino- and carboxy-terminal cleavage fragments (amino acids sequence 1–56, 1–83 and 57–193) were amplified by PCR and subcloned into the pcDNA3-Flag vector. A dually tagged Flag-RhoA-V5-His was also created by introducing a V5-His tag internal to the prenylation sequence at the carboxy-terminal end by PCR.

### Transfection and western blotting

Mouse tissues for Western blotting were collected from C57BL/6 mice. All studies were approved by the McGill University Animal Care and Use Committee. COS-7 cells were transiently transfected for 24h with the different pcDNA3 Flag-RhoA plasmids using Lipofectamine 2000 (Invitrogen, Carlsbad, CA) according to the manufacturer’s instructions. In some experiments, the transfected COS-7 cells were treated for 3h with the calpain inhibitor calpeptin (EMD Millipore/Calbiochem, La Jolla, CA) or with the serine protease inhibitor AEBSF (EMD Millipore/Calbiochem, La Jolla, CA), or for 24h with the pan-caspase inhibitor z-VAD-fmk (R&D Systems, Minneapolis, MN) or the proteasome inhibitors MG132 (Sigma-Aldrich, St-Louis, MO) and epoxomicin (Sigma-Aldrich, St-Louis, MO). For induction of oxidative stress, transfected cells were treated for 1h with increasing doses of hydrogen peroxide (H_2_O_2_) (Thermo Fisher Scientific, Waltham, MA), 24h prior to lysis. Following treatments, the cells were lysed in lysis buffer (50 mM Tris-HCl pH 7.4, 150 mM NaCl, 1 mM EDTA, 1% Triton X-100, 1 mM Na_3_VO_4_, 5 mM NaF, 1X complete protease inhibitors). Sonicated and cleared lysates were loaded in equal amounts of proteins onto SDS-PAGE gels and western blotting was performed with the following antibodies: mouse anti-Flag M2 (Sigma-Aldrich, St-Louis, MO; Catalog #F3165; 1:5000), mouse anti-actin clone C4 (MP Biomedicals, Santa Ana, CA; Catalog #69100; 1:3000), mouse anti-GAPDH (Abcam, Cambridge, UK; Clone 6C5; Catalog #ab8245; 1:2000), mouse anti-c-myc (Sigma-Aldrich, St-Louis, MO; Clone 9E10; Catalog #M5546; 1:2000), rabbit anti-Rho (Abcam, Cambridge, UK; Clone Y486; Catalog #ab32046; 1:500), anti-V5 (Invitrogen, Carlsbad,CA; Catalog #R960-25; 1:5000), and horse-radish peroxidase (HRP)-conjugated anti-mouse and anti-rabbit IgG antibodies (Jackson ImmunoResearch, West Grove, PA; Catalog #111-035-003 and #115-035-003; 1:10000).

### Proteomics

RhoA-NTF and FL-RhoA were immunoprecipitated using Flag M2 agarose beads (Sigma-Aldrich, St-Louis, MO) from lysates prepared from COS-7 cells transiently transfected with pcDNA3 Flag-RhoA Q63L as described above. The proteins were separated by SDS-PAGE and stained with Coomassie Brilliant Blue dye. The bands corresponding to FL-RhoA and -NTF were excised from the gel, digested with either chymotrypsin or trypsin, and submitted to the McGill University and Génome Québec Innovation Centre (Montréal, QC, Canada) for proteomics analysis. The sequences of the peptides generated from these digestions were determined by tandem mass spectrometry and the data analyzed using the Scaffold3 software. To localize the cleavage site, the peptides generated upon trypsin or chymotrypsin digestion and identified by mass spectrometry with 95% probability were aligned onto the theoretical full-length protein sequence of Q63L-RhoA. The regions adjoining but not covered by these peptides and localized in the amino-terminal end of the protein were deemed likely to contain the RhoA cleavage site.

### Protein purification and calpain digestion

Lysates of COS-7 cells transiently transfected with either 2Xmyc WT-RhoA or Flag-RhoA 1–56 were collected in calpain digestion buffer (50 mM Tris-HCl pH 7.4, 100 mM KCl, 1 mM EDTA, 5 mM DTT) without protease inhibitors. Immunoprecipitation was performed with either Flag M2 agarose beads (Sigma-Aldrich, St-Louis, MO) or EZview red anti-c-myc affinity gel (Sigma-Aldrich, St-Louis, MO). Flag-tagged proteins were eluted with 5 μg ml^-1^ Flag peptide (Sigma-Aldrich, St-Louis, MO) in calpain digestion buffer. For *in vitro* digestion, 9 units of recombinant μ-calpain from human erythrocytes (EMD Millipore/Calbiochem, La Jolla, CA) were added to the purified lysates for 45 min at room temperature in the presence of 2 mM CaCl_2_ and the reaction was stopped by the addition of Laemmli sample buffer. As a negative control, 14 μM calpeptin (EMD Millipore/Calbiochem, La Jolla, CA) was added to the samples to inhibit calpain activity.

### Purification of recombinant WT-RhoA

A BL21 *E*. *coli* bacterial culture expressing GST-tagged WT-RhoA was induced overnight at room temperature with 100 μM IPTG. The induced bacteria were pelleted, resuspended in lysis buffer (50 mM Tris-HCl pH 7.4, 50 mM NaCl, 5 mM MgCl_2_, 1 mM DTT, 1X protease inhibitors), and sonicated. The cleared lysate was then incubated with washed glutathione sepharose resin (GE Healthcare, Mississauga, ON) for 2h at 4°C. The beads were washed thoroughly and sequentially with wash buffer I (50 mM Tris-HCl pH 7.4, 150 mM NaCl, 5 mM MgCl_2_, 1 mM DTT) and wash buffer II (50 mM Tris-HCl pH 7.4, 150 mM NaCl, 5 mM MgCl_2_, 2.5 mM CaCl_2_, 1 mM DTT). The beads resuspended in wash buffer II were incubated with 50 units thrombin (Sigma-Aldrich, St-Louis, MO; Catalog #T4648-1kU) overnight at 4°C to elute WT-RhoA without the GST tag. The thrombin-cleaved WT-RhoA was collected in wash buffer III (10 mM Tris-HCl pH 7.4, 2 mM MgCl_2_, 1 mM DTT). The thrombin was removed from the eluate by incubating it with p-aminobenzamidine (PAB) agarose beads (Sigma-Aldrich, St-Louis, MO) at 4°C for 1h. Finally, the eluate was concentrated using Amicon Ultra-4 centrifugal filter units with Ultracel-10 membrane (Millipore, La Jolla, CA). A dose curve of recombinant WT-RhoA was used to quantify the amount of FL-RhoA and NTF-RhoA in some lysates by densitometry with Adobe Photoshop CS5.1. Statistical analysis was done by one-way ANOVA on the data collected from 3 independent experiments.

### Immunoprecipitation of endogenous RhoA-NTF

All studies using rodents were approved by the McGill University Animal Care and Use Committee. Adult Sprague Dawley rats were euthanized by CO_2_ inhalation and cervical dislocation. The brain, heart and lungs were dissected and homogenized in RIPA lysis buffer (50 mM Tris-HCl pH 7.4, 150 mM NaCl, 5 mM EDTA, 1% Triton X-100, 0.1% sodium deoxycholate, 0.1% SDS, 1 mM Na_3_VO_4_, 5 mM NaF, 1X complete protease inhibitors). Alternatively, untransfected COS-7 cells were treated for 1h with 5000 μM H_2_O_2_ and lysed in RIPA lysis buffer 24h following treatment. Cell debris were removed by high-speed centrifugation. The lysates were pre-cleared by incubating them with protein A/G Plus-Agarose beads (SantaCruz Biotechnologies, Santa Cruz, CA) for 1h at 4°C. The endogenous FL-RhoA and -NTF were immunoprecipated from the pre-cleared lysate by incubating it with either 2 μg ml^-1^ anti-Rho antibody (Abcam, Cambridge, UK; Clone Y486; Catalog #ab32046) or anti-GFP antibody (Sigma-Aldrich, St-Louis, MO; Catalog #G1544) as a negative control coupled to A/G agarose beads overnight at 4°C on a rotator. The beads were thoroughly washed in RIPA lysis buffer before eluting the protein by boiling in 2X Laemmli buffer.

### Rhotekin-Binding-Domain (RBD) pull-down

A culture of pGEX2T-RBD *E*. *coli* was induced with 1 mM IPTG for 4h at 30°C after which it was pelleted and resuspended in buffer A (20 mM HEPES pH 7.5, 150 mM NaCl, 5 mM MgCl_2_, 1 mM DTT, 1X complete protease inhibitors). The bacterial lysate was freeze/thawed and sonicated (6X5sec at 20% amplitude with 30s rest). Triton X-100 was added to a final concentration of 0.1% and the lysate was mixed thoroughly before clarification by high-speed centrifugation. The cleared lysate was incubated with glutathione-sepharose beads (GE Healthcare, Mississauga, ON) for 1h at 4°C. The collected GST-RBD beads were washed 3 times with wash buffer (Buffer A containing 0.1% Triton X-100). Beads were freshly prepared before each experiment. In parallel, COS-7 cells were transfected with various pcDNA3 Flag-RhoA mutants using Lipofectamine 2000 (Invitrogen, Carlsbad, CA) following manufacturer’s instructions. The cells were lysed in Mg^2+^ lysis buffer (25 mM HEPES pH 7.5, 150 mM NaCl, 1% NP-40, 10 mM MgCl_2_, 1mM EDTA, 10% glycerol, 1X complete protease inhibitors, 25 mM NaF, 1 mM Na_3_VO_4_). The collected cells were triturated using 25g needles and centrifugated to remove the cell debris. As a positive control for the RBD pull-down, lysate was incubated with GTPgammaS (Millipore, La Jolla, CA) at 30°C for 30min and adding 0.1 M MgCl_2_ ended the reaction. The cell lysates were incubated with the GST-RBD beads for 1h at 4°C. The beads were washed 2 times with Mg^2+^ lysis buffer and eluted using 2X Laemmli buffer.

### Formation of actin stress fibres

Swiss 3T3 fibroblast cells provided by Nathalie Lamarche-Vane (McGill University, Montréal, QC, Canada) were seeded at a density of 75000 cells per well on uncoated 18-mm glass coverslips (Thermo Fisher Scientific, Waltham, MA). After 3 days, Swiss 3T3 cells were serum-starved for 16h and transfected using Lipofectamine LTX and Plus reagent (Invitrogen, Carlsbad, CA) according to the manufacturer’s instructions. To activate both the endogenous and the transfected RhoA proteins, DMEM supplemented with 10% FBS was added to the cells 30 min prior to fixation to induce the formation of actin stress fibres. The cells were fixed with 4% paraformaldehyde (PFA) diluted in phosphate-buffered saline (PBS), permeabilized with 0.2% Triton X-100/PBS and stained with anti-Flag M2 antibody (Sigma-Aldrich, St-Louis, MO; Catalog #F3165; 1:1000) revealed with anti-mouse Alexa-Fluor 488 (Life Technologies/Thermo Fisher Scientific, Waltham, MA; Catalog #A11001; 1:500), Alexa-Fluor 546 Phalloidin (Life Technologies/Thermo Fisher Scientific, Waltham, MA; Catalog #12380; 1:500), and Hoechst dye. The cells were imaged using a Zeiss Axiovert 200M epifluorescence microscope or a Zeiss LSM 710 confocal microscope. Counts were performed to determine the number of transfected cells containing either actin stress fibres (presence of well-defined F-actin filaments within the cytosol) or nuclear actin rods (presence of small actin filaments in the nuclear area) and statistical significance was evaluated using the Chi-square test. In the transfected cells containing stress fibres, the actin accumulation corresponding to fibres was evaluated by measuring the ratio of the actin-covered area divided by the total area of the cell using ImageJ and statistical significance was determined by one-way ANOVA. In those same cells, the orientation of the actin stress fibres was measured using the AngleJ plugin of ImageJ [25]. The data was then organized based on the deviation of the angles from the mode, which corresponds to the angle with the highest number of actin segments. The values were plotted as a frequency distribution and the area under the curve was calculated for a 20° deviation from the mode (0°) to determine the proportion of fibres that exhibited the highest degree of organization.

## Results

### Cleavage of the amino-terminus of RhoA

**Fig 1:**
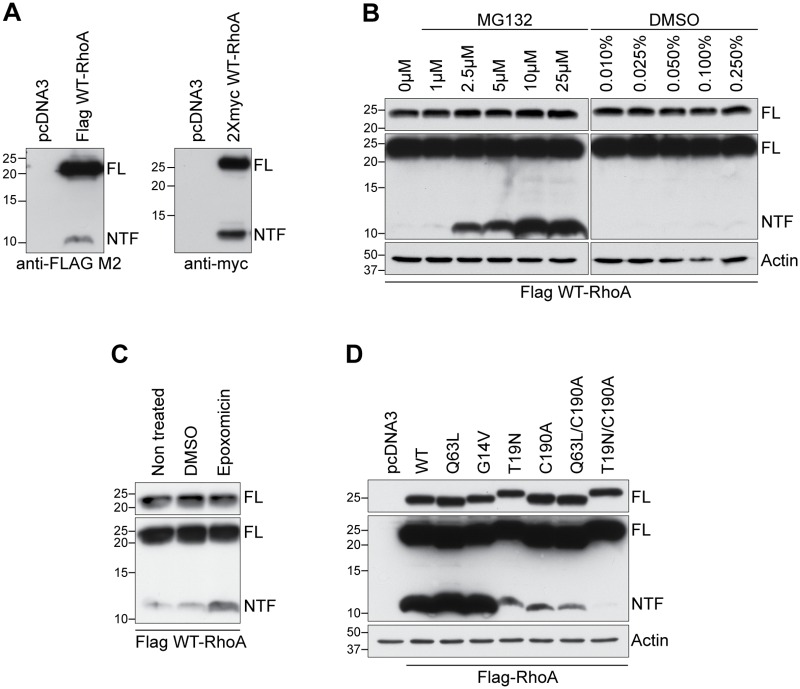
Identification of a 10 kDa amino-terminal RhoA fragment. **A)** Western blot analysis of COS-7 cell lysates transfected with Flag-tagged WT-RhoA or with 2Xmyc-tagged WT-RhoA using an anti-Flag M2 or anti-c-myc antibody reveals the presence of FL-RhoA and RhoA-NTF bands. **B, C)** Western blot of cell lysates following treatment with the proteosome inhibitors MG132 **(B)** or epoxomicin **(C)**. **D)** Expression of wild-type (WT), constitutively active (Q63L and G14V), dominant-negative (T19N) as well as non-prenylated wild-type (C190A), active (Q63L/C190A) and inactive (T19N/C190A) RhoA constructs in COS-7 cells analyzed by western blot using the Flag M2 antibody. Western blot panels illustrating FL-RhoA only are exposed to evaluate loading of full-length protein while panels with FL-RhoA and RhoA-NTF are a longer exposure of the same blot to visualize RhoA-NTF.

Previous reports have shown that calpain and YopT cleave RhoA near its carboxy-terminus to generate a dominant-negative protein [22–24]. We found that overexpression of wild-type (WT) RhoA with a Flag tag on the amino-terminus generated a 22 kDa protein corresponding to full-length RhoA (FL) as well as a 10 kDa proteolytic fragment generated from the amino-terminal end of the protein (RhoA-NTF; Fig 1A). WT-RhoA with a 2Xmyc epitope tag generated a similar fragment indicating that the cleavage is not a function of the epitope tag (Fig 1A). RhoA-NTF was stabilized in cells treated with the proteasome inhibitors MG132 and epoxomicin (Fig 1B and 1C).

### Prenylated active GTP-bound RhoA undergoes enhanced proteolysis

RhoA cycles between an inactive GDP-bound and an active GTP-bound state that interacts with effector proteins to remodel the cytoskeleton [1–3]. To determine whether RhoA proteolysis is dependent on the activation state of the protein, the levels of RhoA-NTF were evaluated in lysates collected from COS-7 cells overexpressing wild-type (WT), constitutively active or dominant-negative RhoA mutants. Constitutively active RhoA (Q63L and G14V) generated higher levels of RhoA-NTF compared to WT-RhoA, whereas these levels were lower with the dominant-negative (T19N) mutant, suggesting that active RhoA is preferentially proteolysed (Fig 1D). In addition to the GDP/GTP cycling, RhoA activity is regulated by prenylation, a post-translational modification essential to its translocation to the plasma membrane [11, 12]. Non-prenylated WT, active and dominant-negative RhoA (C190A-, Q63L/C190A- and T19N/C190A-RhoA, respectively) were weakly proteolysed compared to WT-RhoA (Fig 1D). Together, this illustrates that the proteolytic processing of RhoA occurs preferentially when RhoA is in its prenylated active GTP-bound state.

### Oxidative stress promotes RhoA proteolysis

**Fig 2:**
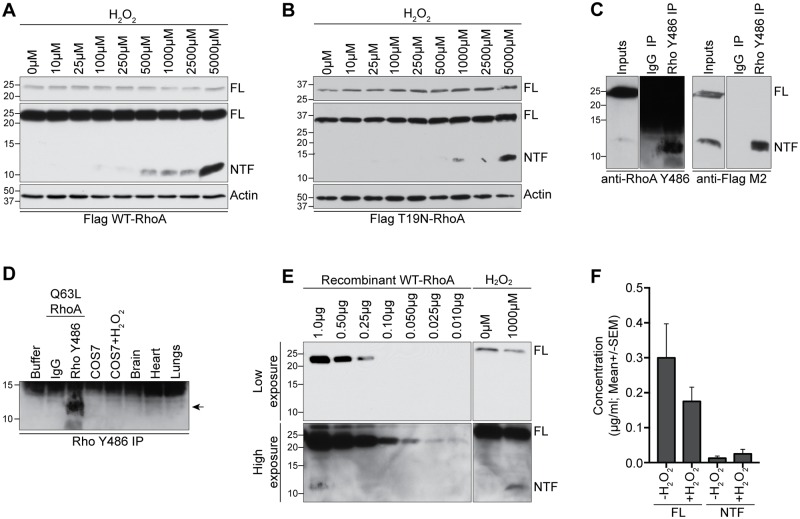
Endogenous RhoA proteolysis is enhanced by oxidative stress. **A, B)** Lysates from COS-7 cells were transfected with Flag-tagged WT-RhoA **(A)** or T19N-RhoA **(B)** and western blotted with anti-Flag M2 antibody. Cells were exposed to H_2_O_2_ for 1 hour, 24 hours prior to lysis. **C)** COS-7 cells transfected with Flag-tagged WT-RhoA were immunoprecipitated with the Rho Y486 antibody from Abcam. Lysates and immunoprecipitates were probed with anti-RhoA Y486 or anti-FLAG M2 antibodies. **D)** Immunoprecipitation of RhoA-NTF from untransfected COS-7 cells treated with H_2_O_2_ for 1 hour at 24 hour prior to lysis or from various healthy adult rat tissues, including the heart, brain and lungs, using the Rho Y486 antibody. Arrow: RhoA-NTF. **E-F)** Analysis of the relative abundance of FL-RhoA and RhoA-NTF in cell lysates compared to a dose curve of recombinant WT-RhoA by Western blot analysis with the Rho Y486 antibody. The graph in F quantifies the average concentration of FL-RhoA as well as RhoA-NTF upon treatment with 1000 μM H_2_O_2_.

Previous studies have shown that RhoA can be activated in response to a number of apoptotic and toxic stimuli [26–29]. To assess whether RhoA proteolysis is altered in response to cell stress, we examined the levels of RhoA-NTF in response to hydrogen peroxide (H_2_O_2_) treatment. Exposure of COS-7 cells expressing either WT- or T19N-RhoA to H_2_O_2_ led to a significant accumulation of RhoA-NTF (Fig 2A and 2B). T19N-RhoA was still subject to H_2_O_2_-dependent proteolysis, indicating that this mechanism is not solely a function of RhoA activation (Fig 2B). To confirm that endogenous RhoA is also subject to proteolytic cleavage, we screened commercially available RhoA antibodies for their ability to detect the RhoA-NTF proteolytic fragment. A single commercial RhoA antibody (clone Y486; Abcam) that was generated against an epitope in the amino-terminal end of RhoA, immunoprecipitates and detects RhoA-NTF from COS-7 cells overexpressing Q63L-RhoA (Fig 2C). The antibody is weak compared to the Flag M2 antibody, as it was able to detect RhoA in the immunoprecipitate but only weakly in the cell lysate (Fig 2C). To determine whether RhoA proteolysis occurs endogenously, we analyzed RhoA immunoprecipitates from untransfected COS-7 cells and detected a weak endogenous RhoA fragment in H_2_O_2_-treated cells (Fig 2D). RhoA-NTF was also primarily detected in lung tissue with weaker bands observed in the heart and brain following immunoprecipitation with the Rho Y486 antibody (Fig 2D). We conclude that endogenous RhoA is proteolytically cleaved but this novel cleavage is difficult to detect because of the weak immunoreactivity of commercial RhoA antibodies.

Using the RhoA Y486 antibody, we quantified the proportion of RhoA-NTF generated in COS-7 cells expressing Flag-tagged WT-RhoA upon H_2_O_2_ treatment. Using densitometry, the intensity of FL-RhoA and RhoA-NTF bands were determined and compared to a dose curve of recombinant WT-RhoA (Fig 2E). In untreated COS-7 cells transfected with WT-RhoA, RhoA-NTF represented on average 5.9% of FL-RhoA, whereas upon treatment with 1000 μM H_2_O_2_, this ratio increased to 17.1% (Fig 2F). Correspondingly, a reduction of 39% in the levels of FL-RhoA was also observed (Fig 2F). We conclude that very little RhoA-NTF is generated in healthy cells and that these levels are appreciably upregulated under conditions of cell stress.

### RhoA proteolysis is regulated by serine proteases, calpain and caspases

**Fig 3:**
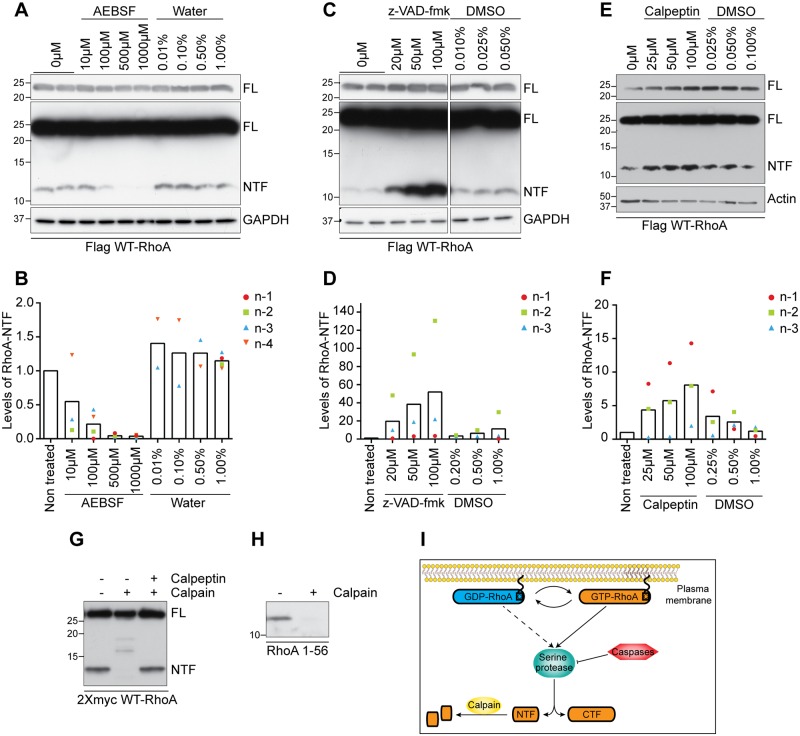
Serine proteases, caspases and calpain regulate RhoA proteolysis. **(A-F)**. Lysates from COS-7 cells transfected with Flag-tagged WT-RhoA were analyzed by western blot with anti-Flag M2 antibody following treatment with protease inhibitors. Transfected cells were treated for 3h with the serine protease inhibitor AEBSF **(A)**, 24h hours with the pan-caspase inhibitor z-VAD-fmk **(C)**, or 3 hours with the calpain inhibitor calpeptin **(E)** and the levels of RhoA-NTF were quantified by densitometry **(B, D, F)**. Water and DMSO were vehicle controls. **(G-H**). 2Xmyc WT-RhoA **(G)** or Flag RhoA 1–56 **(H)** were immunoprecipitated from transfected COS-7 cells and treated for 45 minutes with recombinant μ-calpain in the presence or absence of 14 μM calpeptin. **I)** Diagram illustrating the mechanism underlying RhoA proteolysis.

To identify the protease responsible for the generation of RhoA-NTF, COS-7 cells were transiently transfected with Flag-tagged WT-RhoA and treated with a panel of protease inhibitors. Cells treated with the serine protease inhibitor AEBSF exhibited a dose-dependent decrease in the levels of RhoA-NTF, suggesting that a serine protease is cleaving RhoA (Fig 3A and 3B). Further, a pan-caspase inhibitor (z-VAD-fmk) and a calpain inhibitor (calpeptin) increased the levels of RhoA-NTF in COS-7 cells transfected with WT-RhoA (Fig 3C–3F). These results support a role of calpain and caspases either in the degradation of RhoA-NTF or in the inactivation of the protease responsible for the cleavage. The inability of caspases to directly cleave RhoA supports a model in which caspase regulates a serine protease able to cleave RhoA [30]. In contrast, we find that recombinant μ-calpain directly degrades RhoA-NTF. Treatment of immunoprecipitated 2Xmyc-tagged WT-RhoA or a Flag-tagged amino-terminal fragment of RhoA (1–56) with μ-calpain resulted in the degradation of RhoA-NTF (Fig 3G and 3H). Together, our data suggests that RhoA is cleaved by a serine protease that is targeted and inhibited by caspases, and that RhoA-NTF is directly degraded by calpain (Fig 3I).

### RhoA is cleaved between the Switch I and Switch II regions

**Fig 4:**
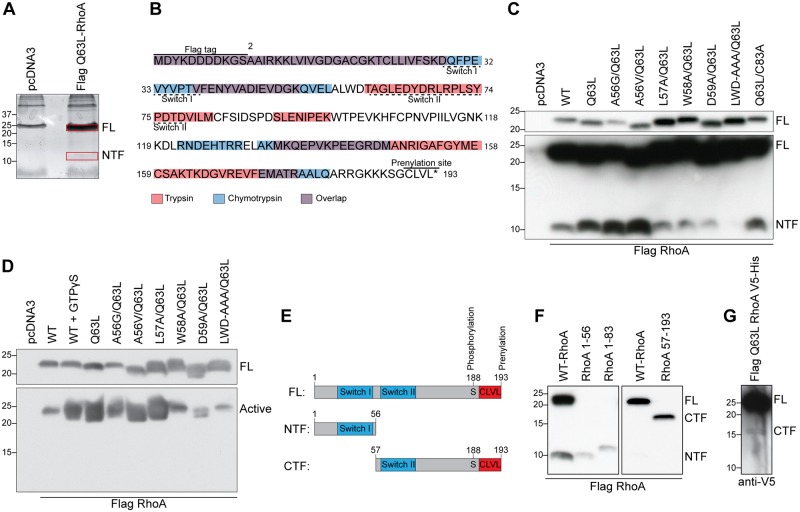
Mapping of the RhoA cleavage site. **A)** Coomassie brilliant blue-stained SDS-Page gel of FL-RhoA and RhoA-NTF showing the bands excised (boxes) for tandem mass spectrometry. **B)** Alignment of the peptides obtained by mass spectrometry of RhoA-NTF following trypsin (red) or chymotrypsin (blue) digest onto the theoretical sequence of Q63L-RhoA. **C)** Mutations were introduced in Q63L-RhoA to localize the cleavage site. **D)** Rhotekin-binding-domain (RBD) pull-down of COS-7 cell lysates transfected with the various Q63L-RhoA mutants evaluated the activation of each construct. **E)** Diagram of the FL-RhoA as well as both amino (NTF)- and carboxy (CTF)- terminal RhoA fragments. **F)** Expression of the constructs encoding the Flag-tagged RhoA fragments illustrated in **E** by western blot with Flag M2 antibody. **G)** Western blot analysis of COS-7 cells lysates transfected with the dual tag Flag Q63L-RhoA V5-His to evaluate the presence of RhoA-CTF using anti-V5 antibody.

To further our understanding of RhoA proteolysis, we sought to map the cleavage site leading to the generation of RhoA-NTF. Flag-tagged Q63L-RhoA was overexpressed in COS-7 cells, and RhoA-NTF was purified from the cell lysate by immunoprecipitation followed by SDS-PAGE separation (Fig 4A). Samples were digested with trypsin or chymotrypsin, and analyzed by tandem mass spectrometry. The peptides obtained with each enzyme were mapped onto the theoretical full-length sequence of Q63L-RhoA. Uncovered regions in the amino-terminal portion of RhoA adjoining detected peptides would be indicative of a potential cleavage site. Unexpectedly, some peptides were identified from the C-terminal region of RhoA and this can likely be ascribed to smear of full length RhoA in the gel. In the amino-terminal region of the protein, potential cleavage sites were identified at residues 56–59 and at residue C83 (Fig 4B). To further investigate the cleavage site, we substituted candidate cleavage residues to Alanine, or to Glycine and Valine in the case of A56 in the Q63L-RhoA background and assessed the sensitivity of mutated constructs to proteolysis. Mutating A56 or C83 had no effect on RhoA proteolysis while mutation at residue L57, W58 or D59 significantly diminished the levels of RhoA-NTF and a combined mutation of these last residues eliminated it (Fig 4C). L57A/Q63L-RhoA is the only mutant with reduced levels of RhoA-NTF that remained active within the cells, as shown by a rhotekin-binding-domain (RBD) pull-down (Fig 4D), suggesting that this construct retains its function within the cell. To characterize the functions of the RhoA cleavage fragments, we generated cDNA constructs encoding residues 1–56 and 57–193, which approximate RhoA-NTF and -CTF, respectively (Fig 4E). Transfection of COS-7 cells with RhoA 1–56 produced a fragment that co-migrated with the one generated upon proteolysis of Flag-tagged WT-RhoA while one encoding residues 1–83 generated a peptide with a molecular weight higher than 10 kDa (Fig 4F). The peptide corresponding to residues 57–193, the predicted carboxy-terminal fragment, generated a 17 kDa protein (Fig 4F). Transfection of a Flag-Q63L-RhoA construct with a V5 tag inserted at the carboxy-terminus preceding the GPI anchor (Flag Q63L-RhoA V5-His) results in expression of a weak V5-positive band at 17 kDa (Fig 4F). The carboxy-terminal RhoA fragment co-migrates with RhoA 57–103 further confirming the proteolytic cleavage site. Weak expression of the carboxy-terminal fragment in cells transfected with full length RhoA suggests that the fragment is unstable in baseline conditions (Fig 4G). These findings support a cleavage site encompassing residues 57–59 localized between the hydrophobic Switch I and Switch II regions [31].

### RhoA-NTF induces mild stress fibre formation while RhoA-CTF leads to the assembly of nuclear actin rods

**Fig 5:**
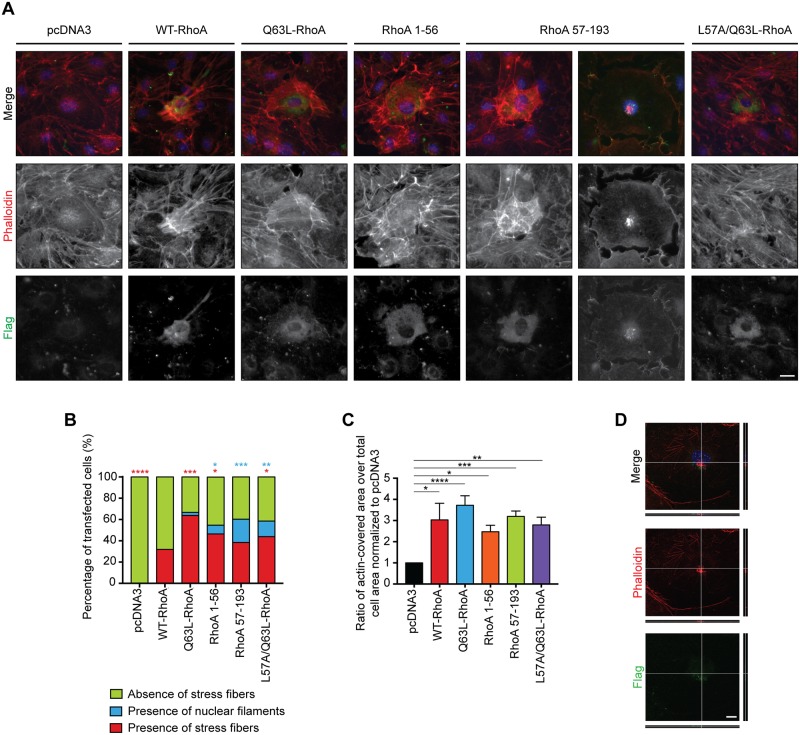
RhoA fragments and cleavage-resistant promote the formation of actin stress fibres. **A)** Serum-starved Swiss 3T3 fibroblasts were transfected with Flag-tagged RhoA 1–56 and 57–193, which correspond to RhoA-NTF and -CTF respectively, as well as WT-, Q63L- and the cleavage-resistant L57A/Q63L-RhoA. Cells were stained with anti-Flag M2 antibody (green), Alexa-Fluor 546 phalloidin (red) and Hoechst (blue) to label RhoA, the actin stress fibres and the nucleus respectively. Scale bar, 20 μm. **B)** Classification of the actin phenotype in transfected Swiss 3T3 cells. Significance was determined by the Chi-square test. *, *p* < 0.05; ** *p* < 0.005; ***, *p* < 0.0005; ****, *p* < 0.0001. *n* > 40 cells from 7 independent experiments. **C)** Quantification of the ratio of actin-covered area divided by the total surface of the transfected cells represented as the mean +/- S.E.M. Significance was established by one-way ANOVA from *n* > 40 cells collected from 7 independent experiments. *, *p* < 0.05; ** *p* < 0.005; ***, *p* < 0.0005; ****, *p* < 0.0001. **D)** Representative side view of a z-stack of a Swiss 3T3 cell overexpressing RhoA-CTF showing the localization of nuclear actin rods (red) relative to the nucleus (blue) and RhoA 57–193 (green). Scale bar, 10 μm.

To investigate the functions of the RhoA fragments generated upon proteolysis, Flag-tagged RhoA 1–56 or 57–193, which correspond to RhoA-NTF and -CTF respectively, was overexpressed in serum-starved Swiss 3T3 fibroblasts and their effects on filamentous actin stress fibres were analyzed. Introduction of either WT- or constitutively active Q63L-RhoA led to the formation of transcytoplasmic actin stress fibres in 31.9% and 63.8% of the transfected cells, respectively (Fig 5A and 5B). This is reflected by a significant increase in the ratio of actin-covered area in the cells containing stress fibres when compared to the cells expressing pcDNA3 (Fig 5C). Flag-tagged RhoA 1–56 and 57–193 also induced the formation of stress fibres in 46.4% and 38.4% of the transfected cells, respectively (Fig 5A and 5B). In both cases, the ratio of actin-covered area was comparable to WT-RhoA, suggesting that a similar amount of actin accumulation occurs in the transfected cells containing stress fibres. Intriguingly, in many cells expressing RhoA 57–193 (38.4%), small actin rods were observed near the nucleus (Fig 5A and 5B). Confocal microscopy revealed that RhoA 57–193 is expressed in close apposition to the actin rods in the proximity of the nucleus (Fig 5D). This suggests that RhoA-CTF could translocate to the nucleus to promote the formation of actin rods and have a potential role in the organization of the nucleoskeleton. Overall, this data indicates that both RhoA-NTF and -CTF retain some activity towards the cytoskeleton by inducing stress fibre formation in cytoplasmic and nuclear regions of the cell.

### RhoA cleavage fragments disrupt the orientation of actin stress fibres induced by endogenous full-length RhoA

**Fig 6:**
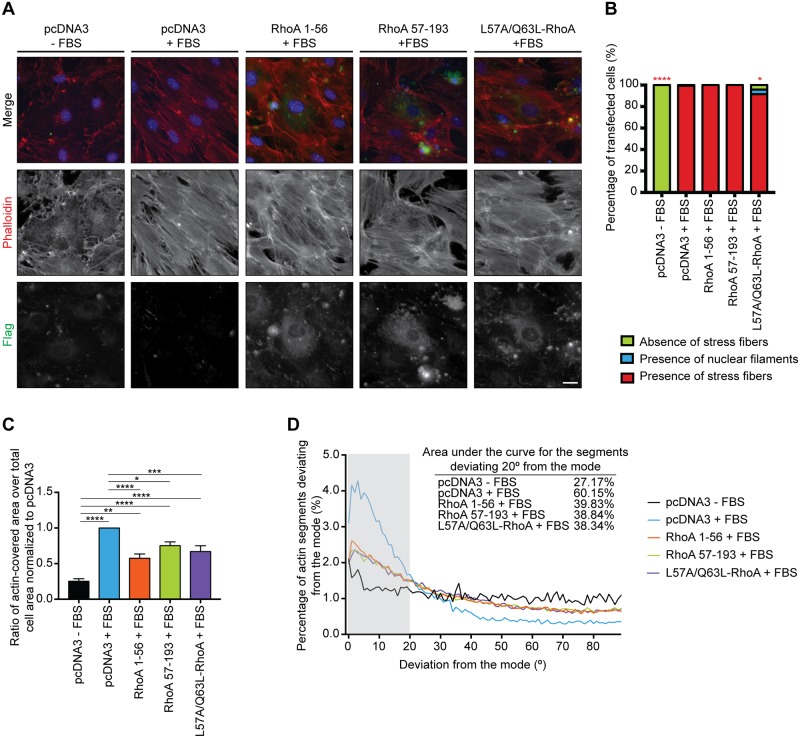
Both RhoA fragments, but not cleavage-resistant RhoA, interfere with the organization of actin stress fibres induced by activated endogenous full-length RhoA. **A)** Serum-starved Swiss 3T3 fibroblasts were transfected the Flag-tagged RhoA 1–56 and 57–193 fragments as well as WT-, Q63L- and L57A/Q63L-RhoA. The endogenous full-length RhoA was activated by treating the cells with 10% FBS for 30 min prior to fixation. The cells were stained with anti-Flag M2 antibody (green), Alexa-Fluor 546 phalloidin (red) and Hoechst (blue) to label RhoA, the actin stress fibres and the nucleus respectively. Scale bar, 20 μm. **B)** Classification of the actin phenotype in the transfected Swiss 3T3 cells. *n* > 20 cells from 3 independent experiments. Significance was determined by the Chi-square test. *, *p* < 0.1; ****, *p* < 0.0001. **C)** Quantification of the ratio of the actin-covered area divided by the total area of the transfected cell represented as the mean +/- S.E.M. Significance was established by one-way ANOVA from *n* > 20 cells from 3 independent experiments. *, *p* < 0.05; ** *p* < 0.005; ***, *p* < 0.0005; ****, *p* < 0.0001. **D)** Frequency distribution of the angles’ deviation from the mode of the actin stress fibres present in the transfected Swiss 3T3 cells as measured with AngleJ. The area under the curve contained within 20° of the mode is representative of the proportion of segments with a high degree of organization.

We next determined whether either RhoA-NTF or -CTF could interfere with the function of endogenous RhoA on actin cytoskeleton remodeling. Swiss 3T3 cells transfected with Flag-tagged RhoA fragments were treated with fetal bovine serum (FBS) to activate RhoA and promote actin stress fibre formation [1]. Cells treated with FBS exhibited long parallel transcytoplasmic actin stress fibres (Fig 6A–6C). In FBS-treated cells the extent of stress fibre formation was diminished in RhoA 1-56- and RhoA 57-193-transfected cells (Fig 6A–6C). Notably, FBS-treated cells transfected with RhoA 1–56 or RhoA 57–193 were characterized by less organized stress fibres. To quantify this phenotype, we quantified the angle of deviation from the mode for all actin segments using AngleJ. The data obtained was then plotted as a frequency distribution with the origin of the graph corresponding to the mode, where the mode represents the orientation that is most common within a given distribution (Fig 6D). Control transfected cells treated with FBS were highly organized with 60% of all actin segments deviating less than 20° from the mode. Cells transfected with RhoA 1–56 or RhoA 57–193 exhibited a more disorganized actin structure with under 40% of transfected cells deviating less than 20° from the mode. We conclude that an effect of the RhoA fragments appears to be a disruption of stress fibre organization upon RhoA activation.

### Proteolysis is not essential to the functions of RhoA towards the actin cytoskeleton

To determine the role of proteolysis in the physiological functions of RhoA towards actin stress fibre formation, we examined the activity of RhoA mutated at residue L57, which is a cleavage-resistant version of this protein that retains RhoA activity (Fig 4C and 4D). This construct was overexpressed in serum-starved Swiss 3T3 fibroblasts to analyze the induction of actin stress fibres. Our data revealed that 43.9% of the transfected cells exhibited stress fibre formation to a level comparable to the one obtained with either WT- or Q63L-RhoA as shown with the ratio of actin-covered area within those cells (Fig 5A–5C). Furthermore, the cleavage-resistant RhoA construct did not interfere with the activity of endogenous WT-RhoA upon activation as 91.5% of transfected cells have long parallel actin stress fibres extending across the cell (Fig 6A and 6B). However, the cells expressing this construct had a reduced ratio of actin-covered compared to the control cells transfected with pcDNA3 and treated with FBS (Fig 6B). The organization of the stress fibres was also impaired (Fig 6D). Thus, cleavage of RhoA is not essential to the induction of actin stress fibres although these are disrupted in their abundance and organization.

## Discussion

Here, we report a novel mechanism of RhoA proteolysis that occurs in the amino-terminal end of the protein to generate a stable 10 kDa fragment. Low levels of endogenous RhoA-NTF are detected in healthy cells and tissues, but these are increased in response to oxidative stress. The mechanism underlying this processing is dependent on a complex interplay of a serine protease, calpain and caspases. RhoA proteolysis is not essential for its activity on the actin cytoskeleton; however RhoA cleavage fragments disrupt the formation of organized stress fibres. RhoA-CTF also promoted the formation of small actin rods near the nuclear area.

### Mechanism underlying RhoA proteolysis

RhoA is a GTPase that acts as a molecular switch, cycling between an inactive GDP-bound and an active GTP-bound state [8, 9]. When transitioning between these states, RhoA undergoes a conformational change in both Switch domains, which exposes the hydrophobic patches necessary to the interaction with downstream effectors [31]. Our findings revealed that RhoA proteolysis occurs preferentially for the active GTP-bound RhoA whereas both inactive and non-prenylated RhoA are poorly cleaved. Additionally, the cleavage site characterized here encompasses residues L57 to D59, which are located next to the Switch II domain. These residues aren’t displaced by the conformational change, but transitioning to the GTP-bound state brings the Switch I domain in close proximity [31]. This could enhance the accessibility of the cleavage site to proteases and explain the increased amount of RhoA-NTF obtained with the constitutively active RhoA. Thus, RhoA proteolysis could be part of a regulation loop that prevents the persistent activation of RhoA.

Furthermore, GTP binding and prenylation favor the translocation of RhoA to the plasma membrane [32, 33], and these, combined with the action of several proteases, regulate RhoA proteolysis. Our data suggest a mechanism where, upon oxidative stress, a serine protease would become activated while caspases would be downregulated, leading to the generation of a stable RhoA-NTF and an unstable RhoA-CTF. Additionally, the proteasome system and calpain would further degrade RhoA-NTF generated upon proteolysis to regulate its stability. The specific serine protease involved in RhoA proteolysis remains to be identified and as such, it is difficult to determine whether it is mediating direct cleavage of RhoA or if it is involved upstream in the regulation of FL-RhoA. Previous reports have documented roles for such proteases in the regulation of RhoA, but these need to be validated for RhoA proteolysis. Potential candidates include the tissue kallikreins-related peptidase (KRK)-5, which reduces the levels of active RhoA via the regulation of isoprenoid synthesis [34], and thrombin since it increases RhoA activity [35]. Additionally, the serine protease Htr2A is upregulated in response to cell stress but its relation to RhoA hasn’t been documented [36]. Similar uncertainties remain concerning the involvement of calpain in RhoA proteolysis as calpeptin, in addition to inhibiting calpain, also promotes RhoA activity by inhibiting a tyrosine phosphatase upstream of this GTPase [37]. Our data support that calpain might be involved in the degradation of RhoA-NTF as it directly cleaves this fragment. However, this does not exclude the activation of RhoA by calpeptin that could lead to the upregulation of RhoA proteolysis. Thus, further experiments are required to characterize the exact role of each protease in RhoA proteolysis.

The constitutive activation of proteases including serine proteases, calpain and caspases is minimal in healthy cells, which could explain the low amount of endogenous RhoA found in healthy cells and tissues. However, oxidative stress leads to high levels of RhoA-NTF, suggesting that it could either activate RhoA or promote its proteolysis. Previous reports have shown that lower doses of H_2_O_2_ induce apoptosis via caspase activation and activate RhoA, whereas higher doses promote necrosis in a caspase-independent fashion [29, 38, 39]. According to our data, the increased levels of RhoA-NTF are observed with the higher doses of H_2_O_2_ associated with necrosis. Furthermore, H_2_O_2_ also promotes proteolysis of a dominant-negative RhoA, suggesting that oxidative stress predominately activates the pathway underlying RhoA cleavage.

### Functions of the RhoA proteolytic fragments

RhoA plays a major role in the reorganization of the actin cytoskeleton by mediating the assembly of stress fibres [1] and this role was confirmed by overexpressing FL-RhoA in Swiss 3T3 fibroblasts. A similar phenotype was observed with the cleavage-resistant RhoA construct although the stress fibres generated were slightly disorganized, but this suggests that proteolysis is not essential for RhoA functions towards the actin cytoskeleton. We next sought to investigate the effects of high levels of RhoA proteolytic fragments. Both RhoA-NTF and RhoA-CTF induces the formation of actin stress fibres, suggesting that these could retain some functions towards the actin cytoskeleton. RhoA effectors are classified into three groups based on their RhoA binding motifs. Class I (eg. rhotekin, PKN, Dia1) and Class III (eg. citron) effectors bind RhoA at a site that partially overlaps with the Switch II and Switch I domains respectively, while Class II (eg. ROCK) effectors interact with RhoA at multiple sites overlapping with both the Switch regions [40]. As the cleavage site is located between the Switch regions, each resulting fragment contains at least one effector-binding domain and should have the ability to interact with RhoA effectors provided that the fragments maintain the appropriate tridimensional conformation. RhoA-NTF contains the binding site for class III effectors and one of the binding sites for class II effectors, whereas RhoA-CTF contains the binding site for class I effectors and two of the three binding sites for class II effectors. Previous reports have shown that both ROCK and Dia1 play a role in actin cytoskeleton remodeling [41], and the ability of the fragments to induce actin stress fibre formation is probably due to the partial interaction with those effectors, but this will need to be confirmed experimentally. Furthermore, both RhoA-NTF and -CTF disrupt the abundance and organization of the actin stress fibres generated by the activation of endogenous RhoA, indicating that they are able to interfere with the functions of full-length RhoA without having a dominant-negative effect. Interestingly, oxidative stress also induces the formation of actin stress fibres, although the mechanism is still poorly characterized [42], but it would be interesting to determine whether RhoA proteolysis is involved in this process.

A striking phenotype was the formation of a network of actin rods and the localization of RhoA-CTF near the nuclear membrane upon its overexpression in fibroblasts. This suggests that this fragment could potentially regulate the nuclear actin cytoskeleton. Under cell stress, actin rods are formed within the nucleus, which could be part of a protective response aiming to minimize the impact of an accumulation of actin within the cytoplasm [43–45]. However, if these nuclear actin rods are maintained, they can impair cellular functions and disrupt nuclear morphology. The presence of nuclear actin rods has been observed in several diseases such as nemaline myopathy [46] and Huntington’s disease [47]. Thus, the generation of RhoA-CTF and its nuclear translocation could either be a protective response to cell stress or a disruption in the nucleoskeleton that could lead to cell death. Further studies are required to investigate the role of RhoA proteolysis in oxidative stress and its link to the formation of nuclear actin rods.

### Proteolysis of other Rho GTPases

The Rho GTPases, including RhoA, Rac and Cdc42, have a high degree of homology in their protein sequences with most of the variations located in the carboxy-terminal end of the proteins [31]. Interestingly, residues L57, W58 and D59 constituting the RhoA cleavage site are conserved between RhoA, RhoB, RhoC, Rac1, and Rac2, while in Cdc42, only residues L57 and D59 are conserved [31]. Thus, it is possible that RhoB, RhoC, Rac1 and Rac2 are also cleaved at this position if the recognition sites for the protease are conserved. As it is well known that crosstalk between the different Rho GTPases occurs during remodeling of the actin cytoskeleton [2, 48], it will be interesting to determine whether regulation through proteolytic cleavage is a mechanism common to multiple Rho GTPases and whether it is partially responsible for this crosstalk. As Rho GTPases are major regulators of the actin cytoskeleton, this novel processing mechanism could potentially be harnessed to regulate cell migration and division, two processes that are essential for normal development as well as central to the progression of cancer.

## Abbreviations

AEBSF: 4-(2-aminoethyl)benzenesulfonylfluoride
BSA: Bovine serum albumin
CaCl_2_: Calcium chloride
DMSO: Dimethyl sulfoxide
DTT: Dithiothreitol
EDTA: Ethylenediaminetetraacetic acid
FBS: Fetal bovine serum
FL-RhoA: Full-length RhoA
GAP: GTPase activating protein
GDI: Guanine nucleotide dissociation inhibitor
GEF: Guanine nucleotide exchange factor
HCl: Hydrogen chloride
H_2_O_2_: Hydrogen peroxide
IPTG: Isopropyl b-D-1-thiogalactopyranoside
KCl: Potassium chloride
MgCl_2_: Magnesium chloride
NaCl: Sodium chloride
NaF: Sodium fluoride
Na_3_VO_4_: Sodium orthovanadate
PBS: Phosphate-buffered saline
PFA: Paraformaldehyde
RBD: Rhotekin-binding domain
RhoA-CTF: Carboxy-terminal RhoA cleavage fragment
RhoA-NTF: Amino-terminal RhoA cleavage fragment
S.E.M.: Standard error of the mean
SDS: Sodium dodecyl sulfate
WT: Wild-type
